# Renal and Blood Pressure Response to a High-Salt Diet in Mice With Reduced Global Expression of the Glucocorticoid Receptor

**DOI:** 10.3389/fphys.2018.00848

**Published:** 2018-07-09

**Authors:** Jessica R. Ivy, Louise C. Evans, Rebecca Moorhouse, Rachel V. Richardson, Emad A. S. Al-Dujaili, Peter W. Flatman, Christopher J. Kenyon, Karen E. Chapman, Matthew A. Bailey

**Affiliations:** University of Edinburgh/British Heart Foundation Centre for Cardiovascular Science, The Queen’s Medical Research Institute, The University of Edinburgh Medical School, The University of Edinburgh, Edinburgh, United Kingdom

**Keywords:** hypertension, renal, sodium, steroids, aldosterone, thiazide, ENaC, mineralocorticoid

## Abstract

Salt-sensitive hypertension is common in glucocorticoid excess. Glucocorticoid resistance also presents with hypercortisolemia and hypertension but the relationship between salt intake and blood pressure (BP) is not well defined. GR^βgeo/+^ mice have global glucocorticoid receptor (GR) haploinsufficiency and increased BP. Here we examined the effect of high salt diet on BP, salt excretion and renal blood flow in GR^βgeo/+^mice. Basal BP was ∼10 mmHg higher in male GR^βgeo/+^ mice than in GR^+/+^ littermates. This modest increase was amplified by ∼10 mmHg following a high-salt diet in GR^βgeo/+^ mice. High salt reduced urinary aldosterone excretion but increased renal mineralocorticoid receptor expression in both genotypes. Corticosterone, and to a lesser extent deoxycorticosterone, excretion was increased in GR^βgeo/+^ mice following a high-salt challenge, consistent with enhanced 24 h production. GR^+/+^ mice increased fractional sodium excretion and reduced renal vascular resistance during the high salt challenge, retaining neutral sodium balance. In contrast, sodium excretion and renal vascular resistance did not adapt to high salt in GR^βgeo/+^ mice, resulting in transient sodium retention and sustained hypertension. With high-salt diet, *Slc12a3* and *Scnn1a* mRNAs were higher in GR^βgeo/+^ than controls, and this was reflected in an exaggerated natriuretic response to thiazide and benzamil, inhibitors of NCC and ENaC, respectively. Reduction in GR expression causes salt-sensitivity and an adaptive failure of the renal vasculature and tubule, most likely reflecting sustained mineralocorticoid receptor activation. This provides a mechanistic basis to understand the hypertension associated with loss-of-function polymorphisms in GR in the context of habitually high salt intake.

## Introduction

Salt-sensitive blood pressure (BP) is an independent risk factor for both cardiovascular and renal disease and the salt-induced rise in BP reflects underlying renal (Hall, 2016) and vascular (endothelial) dysfunction (Morris et al., 2016). The failure of neural and hormonal regulatory systems to adapt to high dietary salt intake is an important contributing factor to salt-sensitivity. In this context, sustained glucocorticoid excess, whether overt in Cushing’s or covert in Metabolic Syndrome, induces salt-sensitive hypertension and increases cardiovascular risk (Bailey, 2017).

How glucocorticoids induce salt-sensitive hypertension is not clearly understood. Several groups, including ours, have used animal models to examine the relationship between abnormal glucocorticoid activity and BP. For example, both modest glucocorticoid excess (Bailey et al., 2009; Mu et al., 2011) and impaired glucocorticoid metabolism (Bailey et al., 2011; Craigie et al., 2012; Mullins et al., 2015) cause salt-sensitive hypertension. Abnormal activation of the thiazide-sensitive cotransporter (NCC) (Velazquez et al., 1996; Ivy et al., 2016) and the epithelial sodium channel (ENaC) (Gaeggeler et al., 2005; Bailey et al., 2009) both contribute to the rise in BP. More subtly, clamping corticosterone in the mid-physiological range ablates the normal diurnal rhythmicity, activates NCC and induces non-dipping BP in mice (Ivy et al., 2016). Interestingly, adrenalectomy also induces non-dipping BP in mice (Sei et al., 2008) and in humans such circadian abnormalities have a detrimental impact on cardiovascular health (Henley and Lightman, 2014).

In the current study, we turned our focus from models of glucocorticoid excess to a model of glucocorticoid resistance. In humans, glucocorticoid resistance (OMIM 615962) induces hypercortisolemia but patients are not Cushingoid due to loss of function mutations in NR3C1, the gene encoding the glucocorticoid receptor (GR). In this study, we used a transgenic mouse where *Nr3c1* was disrupted by gene-trap integration of a β^geo^ reporter. This created a fusion protein lacking the ligand binding domain and thus rendered transcriptionally inactive (Michailidou et al., 2008). Homozygous transgenic mice (GR^βgeo/βgeo^) die postnatally but heterozygous transgenics (GR^βgeo/+^) survive to adulthood and mRNA expression for GR in the brain, adrenal gland, liver, fat and muscle is reduced by 40–50% (Michailidou et al., 2008). In that study, mice were fed rodent chow containing 0.25% sodium. In humans, daily sodium intake habitually exceeds the recommended upper limits (Powles et al., 2013) and the interaction between dietary salt, genetic abnormalities in GR and BP homeostasis is not well defined. In this study, we therefore exposed GR^βgeo/+^ mice to a high salt diet, revealing salt-sensitive hypertension and renal tubular and vascular abnormalities.

## Materials and Methods

Mice were backcrossed onto the C57BL/6J background for >8 generations. In the current study, all experiments were performed on male GR^βgeo/+^ mice aged 12–16 weeks using GR^+/+^ littermates as controls. Experiments were performed under a UK Home Office License, following ethical review by the University. Mice were fed either a standard diet (0.25% Na, RM1) or a high-sodium diet (2.5% Na diet 829504), both manufactured by Special Diets Services Ltd. (Essex, United Kingdom). All materials were obtained from Sigma-Aldrich UK unless otherwise stated.

### Blood Pressure

**FIGURE 1:**
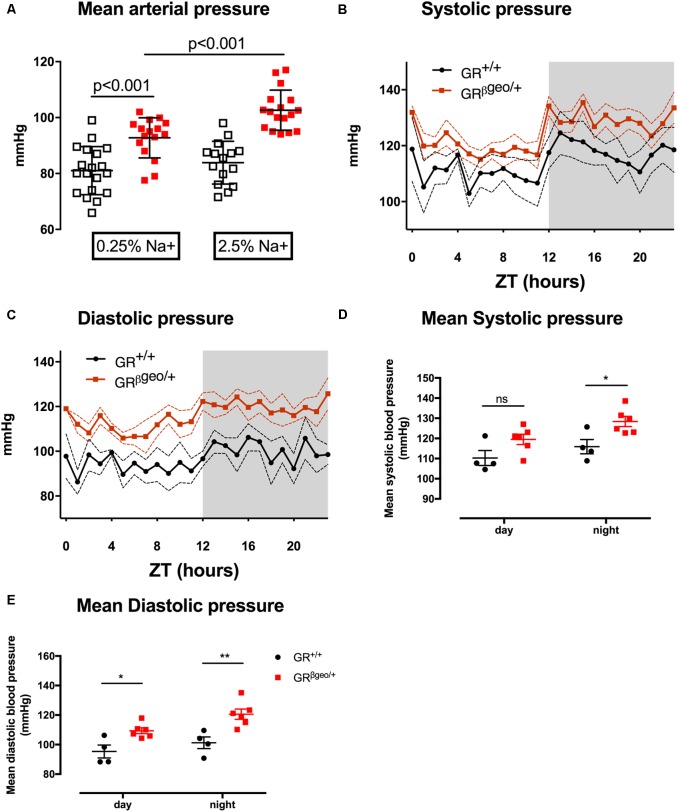
Mean arterial pressure **(A)** was measured acutely in GR^+/+^ (open squares) or GR^βgeo/+^ (red squares) littermates. Mice were fed either 0.25% sodium (*n* = 19/15) or 2.5% sodium diet (*n* = 15/17). Data are provided for individual mice, along with mean ± SEM for each group, and comparisons were made by two-way ANOVA with *post hoc* Holm–Šidák tests for defined comparisons. **(B,C)** In a second group of conscious, unrestrained mice, blood pressure was measured by radiotelemetry. GR^+/+^ (black line; *n* = 4) and GR^βgeo/+^ (red line; *n* = 6), mice. Data are group mean generated by averaging each mouse over 2 consecutive days of recording, dotted lines indicate SEM. ZT = 0 was 0700 h local time. Lights were on from ZT 0–12 and mice were asleep during this subjective day. The shaded area represents the period of subjective night, when mice were active. **(D,E)** 12-hour mean day/night systolic and diastolic pressure is provided for individual mice along with mean ± SEM for each group and comparisons made with two-way ANOVA with *post hoc* Holm–Šidák tests for defined comparisons. ^∗^*p* < 0.05 and ^∗∗^*p* < 0.01.

Blood pressure was measured in a cross-sectional study in mice fed 0.25% Na diet (GR^βgeo/+^ *n* = 16; GR^+/+^ *n* = 19) or 2.5% Na diet (GR^βgeo/+^ *n* = 15; GR^+/+^ *n* = 17). The carotid artery was cannulated under thiobutabarbital anesthesia (Inactin; 120 mg/kg IP) and after a 40 min stabilization period, mean arterial BP was recorded over 30–40 min and an average taken for each mouse. In a separate cohort of mice, BP under basal conditions (0.25% Na intake) was measured by radiotelemetry in GR^βgeo/+^ (*n* = 6) and control (*n* = 4) mice, as described (Ivy et al., 2016). Mice were anesthetized with isoflurane (2% in O_2_ for induction; 1.5% for maintenance; IsoFlo, Zoetis UK Limited, United Kingdom) and PAC-10 radiotelemeter devices (Data Sciences International, United States) were inserted with the catheter placed in the carotid artery. After a 7-day recovery period, systolic blood pressure (SBP) and diastolic blood pressure (DBP) were recorded for a 1 min period every 60 min at an acquisition rate of 1 kHz. The data are presented in two ways. First, to give a view of blood pressure through the 24 h cycle, we took an average for each time point for each mouse over 2 consecutive days of recording and used these data to calculate the group mean shown in **Figures 1B,C**, as described (Evans et al., 2016). Second, for each mouse we took a 12 h average, making between genotype comparisons for SBP and DBP (**Figures 1D,E**).

### Sodium Balance in Conscious Mice

Mice (*n* = 6 per genotype) were housed in metabolic cages with free access to food and water, intake of which was measured daily. Mice were fed 0.25% Na chow for 3 days and then 2.5% Na chow for 6 days. Food intake was used to calculate daily sodium intake. Urine and feces were collected every 24 h and the sodium content of each was combined to provide daily sodium output. Flame photometry (BWB Technologies, United Kingdom) was used to measure sodium concentration in urine and the sodium content in dry feces following extraction in nitric acid (∼15 M; Thermo Fisher Scientific, United Kingdom), as described (Christensen et al., 2015). Cumulative sodium balance (intake-output) was calculated over periods of 3 days. In-house, validated enzyme-linked immunosorbent assays were used to measure urinary concentration of aldosterone (Al-Dujaili et al., 2009b), corticosterone and deoxycorticosterone (Al-Dujaili et al., 2009a). For each mouse, measurements were made on urine collected on day 1 and day 3 (baseline) and day 5 and day 6 (high salt). Measurements were averaged used to calculate the group means. 24 h urinary steroid excretion was used as an index of global activity of the renin–angiotensin–aldosterone system and hypothalamic-pituitary-adrenal axis.

### Renal Function

GR^βgeo/+^ and GR^+/+^ wild-type mice were maintained on either a 0.25 or 2.5% Na diet for 7 days and then anesthetized (Inactin; 120 mg/kg; IP) and prepared surgically for renal clearance experiments. Experiments were performed between 9 am and 5 pm local time. Previous work indicates that under Inactin anesthesia renal function is similar to that measured in conscious rodents during the active phase (Walter et al., 1989). The carotid artery was cannulated for sampling of arterial blood and the bladder was catheterized for collection of urine. The jugular vein was cannulated for infusion of a solution containing 120 mmol/l NaCl, 15 mmol/l NaHCO_3_ and 5 mmol/l KCl. FITC-conjugated inulin (0.25% w:v) and *p*-aminohippurate (sodium salt, 20 mmol/l) were included in the infusate to allow the measurement of glomerular filtration rate (GFR) and renal blood flow (RBF) by clearance methodology, as described (Hunter et al., 2014). After a post-surgery stabilization period, a baseline urine collection was made and then each mouse received an injection of either hydrochlorothiazide (2 mg/kg; IV) or benzamil (1 mg/kg IV). After 20 min, a second urine collection was made. Urine and plasma sodium concentration was measured by ion-selective electrode (9180 Electrolyte Analyzer, Roche UK) and used to calculate fractional sodium excretion (FENa), expressed as the percentage of the filtered sodium load excreted in the urine. The net natriuretic response (ΔFENa) to thiazide was used as an index of NCC activity; that of benzamil as an index of ENaC activity, as described (Hunter et al., 2014).

### Quantitative PCR

GR^+/+^ and GR^βgeo/+^ mice were maintained on either a 0.25 or 2.5% sodium diet as above. Following cervical dislocation, the kidneys were snap frozen and mRNA later extracted using Trizol (Invitrogen, United Kingdom) and a Purelink RNA mini kit (Qiagen, United States) and quantified using a NanoDrop 1000 spectrophotometer (Thermo Fisher Scientific, United Kingdom). RNA concentrations were 200–700 ng/μl and the A260/280 ratios were >1.7. mRNA abundance was assessed by quantitative PCR. In the first experiment, the expression of renal *Nr3c1*, *Nr3c2*, and *Hsd11b2* quantified. In the second experiment the expression of *Scnn1a* and *Slc12a3* were measured. Assays were developed using the Universal Probe Library and performed on a LightCycler 480 (both from Roche, United Kingdom). Each sample was assessed in triplicate and an average value taken. *Tbp* was not different by either diet or genotype and was used here as a control reference gene and all other value were normalized to this expression. qPCR data are expressed relative to GR^+/+^ expression under basal conditions, the group mean of which was set arbitrarily to 100%. The probe number and primer sequences are shown in **Table 1**.

**Table 1:** Primers for qPCR.

Gene	Forward sequence	Reverse sequence	Probe number
*Tbp*	gggagaatcatggaccagaa	gatgggaattccaggagtca	97
*Nr3c1*	caaagattgcaggtatcctatgaa	cttggctcttcagaccttcc	91
*Nr3c2*	caaaagagccgtggaagg	tttctccgaatcttatcaataatgc	11
*Hsd11b2*	cactcgaggggacgtattgt	gcaggggtatggcatgtct	26
*Slc12a3*	cctccatcaccaactcacct	ccgcccacttgctgtagta	12
*Scnn1a*	ccaagggtgtagagttctgtga	agaaggcagcctgcagttta	45

*The genes encode the following proteins: TATA-binding protein, glucocorticoid receptor, mineralocorticoid receptor, 11 β-hydroxysteroid dehydrogenase type 2, the thiazide-sensitive co-transporter and the alpha subunit of the Epithelial Sodium Channel.*

### Statistics

Data are presented as mean ± standard error or as medians with interquartile range, as appropriate. Statistical comparisons (GraphPad Prism 7, GraphPad Software, La Jolla, CA, United States) were made by using two-way analysis of variance to assess the main effects of the genotype and diet and the interaction between the main effects. Post-test comparisons were made by using the Holm–Šidák test for multiple comparisons. For sodium and water balance, the non-parametric Kruskal–Wallis test was used, with post-test comparisons being made with Dunn’s tests. A *p*-value of <0.05 was considered statistically significant.

## Results

### GR^βgeo/+^ Mice Have Salt-Sensitive Hypertension

Blood pressure was ∼10 mmHg higher in anesthetized GR^βgeo/+^ mice than in GR^+/+^ mice under normal salt diet of 0.25% sodium (**Figure 1A**). This was confirmed in a separate group of conscious, unrestrained mice by radiotelemetry. SBP (**Figures 1B,D**) was significantly higher (*p* < 0.05) only during the subjective night; DBP (**Figures 1C,E**) was significantly higher in both day (*p* < 0.05) and night (*p* < 0.01) periods. BP was next measured in groups of anesthetized mice maintained on a high (2.5% Na) salt diet for 6 days. There was no effect of salt on BP in GR^+/+^ mice, indicating salt-resistance. In contrast, high salt increased BP by ∼10 mmHg in GR^βgeo/+^ mice (**Figure 1A**): there was a significant effect of salt (*p* < 0.0001), genotype (*p* < 0.001) and a significant interaction (*p* < 0.05 interaction).

**FIGURE 2:**
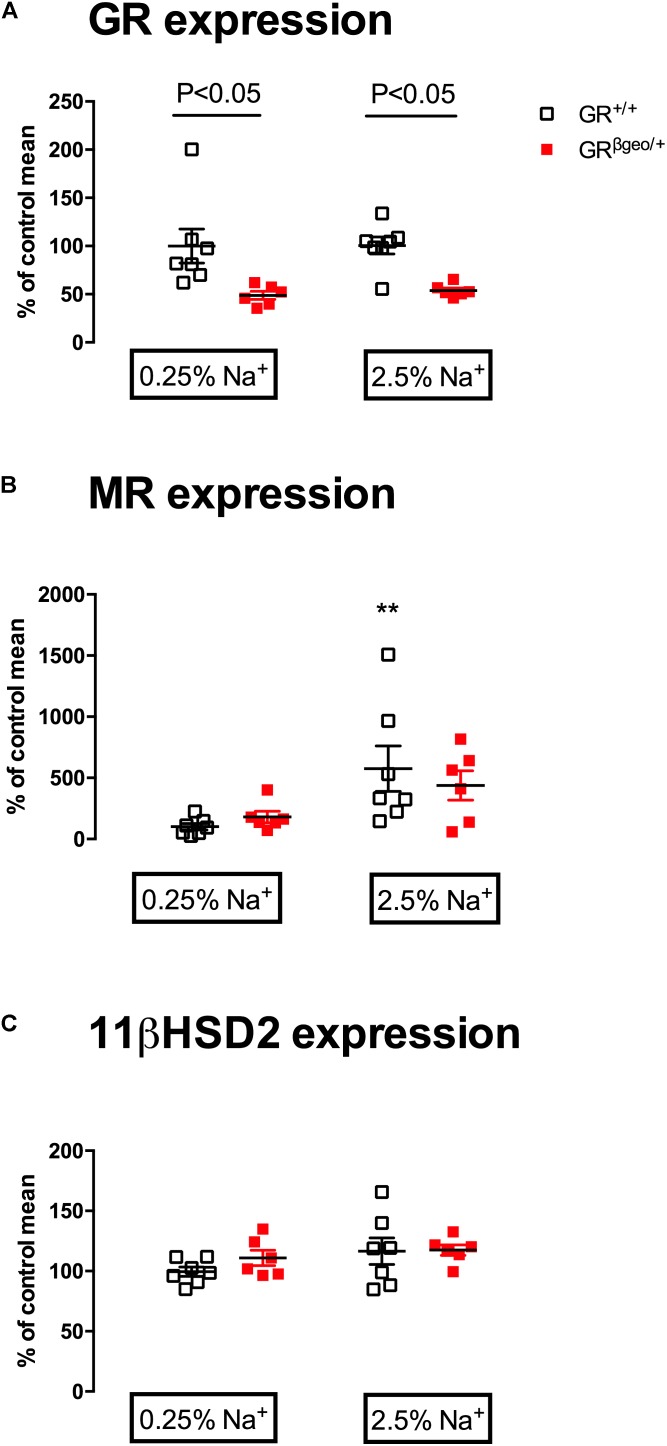
mRNA expression of **(A)** *Nr3c1*, encoding GR; **(B)** *Nr3c2*, encoding MR; and **(C)** *Hsd11b2*, encoding 11β-hydroxysteroid dehydrogenase type 2 (11βHSD2) in GR^+/+^ (open squares; *n* = 7/7) and GR^βgeo/+^ mice (red squares; *n* = 6/6) on 0.25% sodium or a 2.5% sodium diet. Data from individual mice are presented, along with the group mean ± SEM. Data were analyzed by two-way ANOVA with *post hoc* Holm–Šidák tests. Post-test comparisons of the effect of a high salt within genotype are shown ^∗∗^*p* < 0.01. Between-genotype post-test comparisons are as indicated.

Using quantitative PCR we demonstrated that GR^βgeo/+^ mice had ∼50% reduction in renal *Nr3c1* (encoding GR) mRNA (**Figure 2A**; Effect of genotype *p* < 0.001). There was no significant effect of 6-days of high salt diet on renal *Nr3c1* expression. There was also no significant effect of genotype on the renal expression of *Nr3c2* (encoding MR) but dietary salt increased expression significantly (*p* < 0.01) and to a similar extent in both groups of mice (**Figure 2B**). The expression of *Hsd11b2*, which encodes the glucocorticoid metabolizing enzyme 11β-hydroxysteroid dehydrogenase type 2 (11βHSD2), was not different between groups and was not affected by high salt diet (**Figure 2C**).

### GR^βgeo/+^ Mice Show Transient Sodium Retention

**FIGURE 3:**
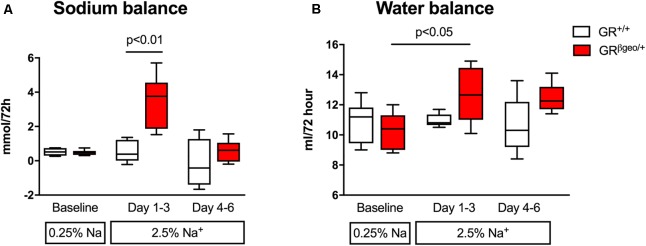
Sodium **(A)** and water balance **(B)** in GR^+/+^ (*n* = 5; open boxes) and GR^βgeo/+^ (*n* = 6; red boxes) mice on a 0.25% sodium diet and then a 2.5% sodium diet. Cumulative sodium and water balance is displayed in 3-day bins at baseline (first pair of bars) and during adaptation to a high sodium diet (the second pair of bars represent Na balance over days 1-3; the third set days 4–6 of high sodium feeding). Data are median and range and were analyzed by non-parametric Kruskal–Wallis tests, with *post hoc* Dunn’s tests.

Sodium balance was measured over periods of 3 days, neutral balance being confirmed in both genotypes before the high salt challenge. GR^βgeo/+^ mice entered positive sodium balance over the first three days of 2.5% Na diet (**Figure 3A**), regaining neutral sodium balance during the second 3-day period of high salt. Wild-type mice remained in neutral sodium balance throughout the 6 days of high salt diet (**Figure 3A**). Water balance followed the same profile as that of sodium: GR^βgeo/+^ mice retained more fluid than wild-types in the first 3 days of the high salt challenge (**Figure 3B**) and tended to retain more water during days 4–6, but this was not statistically significant.

### High Salt Diet Increases Corticosterone Excretion in Wild Type and GR^βgeo/+^ Mice

**FIGURE 4:**
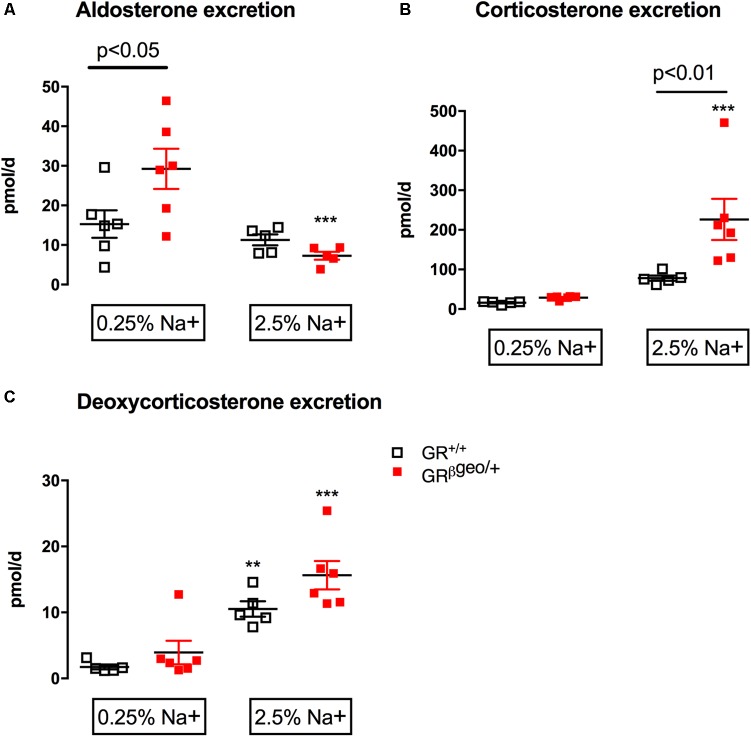
Twenty-four hours urinary excretion of **(A)** aldosterone, **(B)** corticosterone, and **(C)** deoxycorticosterone in GR^+/+^ (*n* = 5; open squares) and GR^βgeo/+^ (*n* = 6; red squares) mice on 0.25% sodium and then 2.5% Na diet. The 3-day average for each mouse is shown along with the group mean ± SEM. Data were analyzed by two-way ANOVA with *post hoc* Holm–Šidák tests. Post-test comparisons of the effect of a high salt within genotype are shown as ^∗^*p* < 0.05, ^∗∗^*p* < 0.01, ^∗∗∗^*p* < 0.001. Between-genotype post-test comparisons are as indicated.

On 0.25% sodium diet, urinary aldosterone excretion was significantly higher in GR^βgeo/+^ mice than in controls (**Figure 4A**; Effect of genotype *p* < 0.05). High salt feeding suppressed aldosterone excretion in both groups (Effect of salt *p* < 0.01) and excretion was not different between genotypes in this phase of the experiment (**Figure 4A**). Basal corticosterone excretion was higher in GR^βgeo/+^ mice (29 ± 2 pmol/day) than GR^+/+^ (14 ± 1 pmol/day) but this difference was not statistically significant. High dietary salt significantly increased urinary corticosteroid excretion in both GR^+/+^ and GR^βgeo/+^ mice (Effect of salt *p* < 0.001), with the effect being more exaggerated in GR^βgeo/+^ mice (**Figure 4B**; Effect of genotype, *p* < 0.05; Interaction *p* < 0.05). Excretion of deoxycorticosterone, which is a corticosterone precursor with weak mineralocorticoid activity, was significantly enhanced by dietary salt in both groups of mice (**Figure 4C**; Effect of salt *p* < 0.001), again with a larger effect observed in GR^βgeo/+^ mice (Effect of genotype *p* < 0.05).

### GR^βgeo/+^ Mice Have Impaired Renal Hemodynamic and Tubular Adaptation to High Salt

**FIGURE 5:**
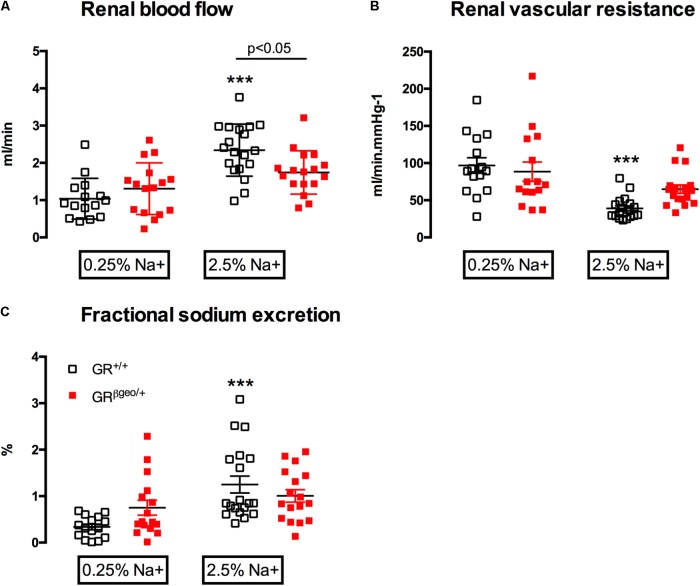
**(A)** Renal blood flow, **(B)** renal vascular resistance, and **(C)** fractional sodium excretion in GR^+/+^ (open squares) and GR^βgeo/+^ (red squares) mice maintained on either a 0.25% sodium or 2.5% Na diet. Data from individual mice are presented as are the group mean ± SEM. Data were analyzed by two-way ANOVA with *post hoc* Holm–Šidák tests. *Post hoc* comparisons of the effect of a high salt within genotype are shown ^∗∗∗^*p* < 0.001. Between-genotype *post hoc* comparisons are as indicated.

Renal clearance experiments were performed on GR^βgeo/+^ and wild-type mice that had been fed either control or high salt diet for 6 days prior to anesthesia and surgery. Glomerular filtration rate was similar between genotypes and not affected by dietary salt (**Table 2**). Under basal conditions renal blood flow (**Figure 5A**) and renal vascular resistance (**Figure 5B**) did not differ between genotypes. High salt diet doubled renal blood flow in GR^+/+^ mice (**Figure 5A**; Effect of salt *p* < 0.001); the hyperemic effect was attenuated in GR^βgeo/+^ mice and did not achieve statistical significance. Thus, RBF was significantly lower in high salt-fed GR^βgeo/+^ mice compared to GR^+/+^ controls (**Figure 5A**; Interaction *p* < 0.01) and renal vascular resistance, which was reduced by high salt in GR^+/+^ mice, did not change significantly in GR^βgeo/+^ mice (**Figure 5B**).

**Table 2:** Glomerular Filtration Rate (GFR), plasma sodium and potassium concentration and hematocrit in GR^+/+^ and GR^βgeo/+^ mice maintained on either 0.25% (*n* = 15/16) or 2.5% sodium diet (*n* = 17/19) for 7 days.

	0.25% Na^+^		2.5% Na^+^	
	GR^+/+^	GR^βgeo/+^	GR^+/+^	GR^βgeo/+^
GFR (ml/min)	0.20 ± 0.02	0.20 ± 0.02	0.25 ± 0.02	0.22 ± 0.02
Plasma Na (mmol/l)	145.7 ± 1.0	144.2 ± 0.9	145.3 ± 0.9	144.6 ± 1.1
Plasma K (mmol/l)	4.4 ± 0.2	4.2 ± 0.1	4.5 ± 0.2	4.5 ± 0.2
Hematocrit (%)	42.7 ± 1.0	45.0 ± 0.9^∗∗∗^	44.0 ± 0.3	46.8 ± 0.7^∗∗∗^

*Data are mean ± SEM and were analyzed by two-way ANOVA with post hoc Holm–Šidák tests, ^+^p < 0.05, ^+++^p < 0.001 significant effect of high salt diet within a given genotype, ^∗∗∗^p < 0.001 ^∗∗^p < 0.01 significant effect of genotype within a given diet.*

FE_Na_ tended to be higher in GR^βgeo/+^ mice than in GR^+/+^ mice on a 0.25% sodium diet, but this was not significantly different. High salt diet increased FE_Na_ in GR^+/+^ mice (**Figure 5C**), indicating appropriate downregulation of tubular sodium reabsorption. In contrast, GR^βgeo/+^ mice, failed to modulate tubular sodium transport in response to high salt and FE_Na_ was not significantly increased (**Figure 5C**; Effect of salt *p* < 0.001; interaction; *p* < 0.05). Impaired renal sodium excretion was not associated with a change in plasma sodium or potassium (**Table 2**). Hematocrit was significantly higher in GR^βgeo/+^ mice than controls but there was no effect of salt diet in either genotype (**Table 2**). This is difficult to interpret in terms of relative volume status since GR has an important role in erythropoiesis (Bauer et al., 1999).

### GR^βgeo/+^ Mice Do Not Downregulate NCC or ENaC in Response to Salt Loading

To investigate the molecular mechanisms of altered renal sodium excretion, we focused on the distal nephron, first measuring the mRNA levels of NCC and ENaC, then using the inhibitors thiazide and benzamil to assess protein function *in vivo*.

**FIGURE 6:**
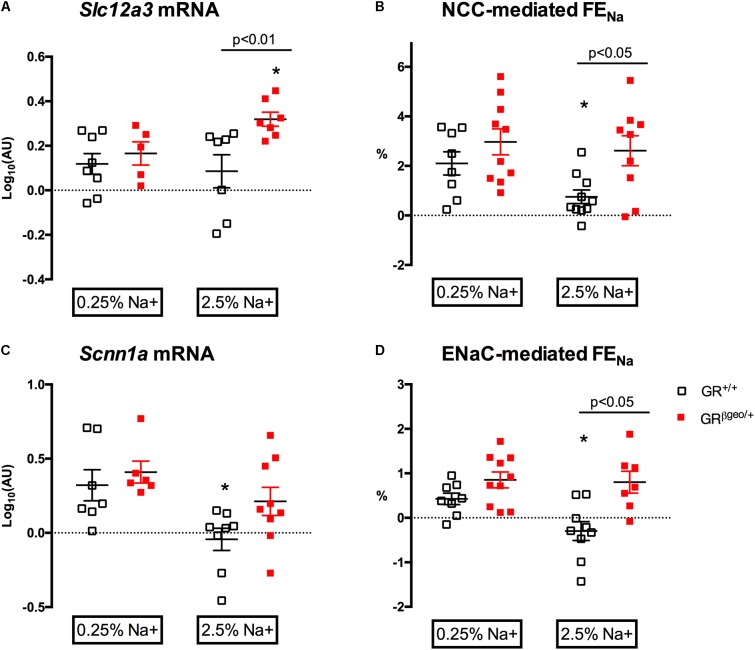
Sodium transporter mRNA abundance and *in vivo* activity in GR^+/+^ (open squares) and GR^βgeo/+^ mice (red squares) on 0.25% sodium or a 2.5% sodium diet. **(A)** mRNA expression of *Slc12a3*, encoding NCC; **(B)** the net natriuretic response to thiazide, taken to reflect NCC-mediated sodium reabsorption; **(C)** mRNA expression of *Scnn1a*, encoding α subunit of ENaC; **(D)** the net natriuretic response to benzamil, taken to reflect ENaC-mediated sodium reabsorption. AU, arbitrary units. Data from individual mice are presented, along with the group mean ± SEM. Data were analyzed by two-way ANOVA with *post hoc* Holm–Šidák tests. Post-test comparisons of the effect of a high salt within genotype are shown ^∗^*p* < 0.05. Between-genotype post-test comparisons are as indicated.

*Slc12a3* mRNA abundance was comparable between genotypes on a standard sodium diet (**Figure 6A**) and thiazide increased FE_Na_ by 2-3% in both genotypes (**Figure 6B**), consistent with our previous estimates of *in vivo* NCC transport in C57BL/6 mice (Hunter et al., 2014). A 6-day high salt diet did not change *Slc12a3* mRNA expression in GR^+/+^ mice, but thiazide-sensitive sodium transport was significantly reduced, indicating downregulation of functional NCC. In GR^βgeo/+^ mice, high salt feeding significantly increased *Slc12a3* mRNA (**Figure 6A**): the net-natriuretic response to the thiazide did not increase but was sustained, not downregulated, in high salt-treated GR^βgeo/+^ mice. Overall, the two genotypes had significantly different a natriuretic response to thiazide (Effect of genotype *p* < 0.01).

The expression level of *Scnn1a* mRNA, encoding the alpha subunit of ENaC, did not differ between genotypes (**Figure 6C**) on 0.25% Na diet. The natriuretic response to benzamil was similar in both groups and indicated that under control salt conditions, ENaC reabsorbed ∼0.5% of the filtered sodium load (**Figure 6D**), consistent with our previous studies for this mouse strain (Bailey et al., 2009). In GR^+/+^ mice, 6 days of high salt diet caused a down-regulation of *Scnn1a* mRNA (**Figure 6C**) and abolished benzamil-sensitive sodium reabsorption (**Figure 6D**). This correlation of transcriptional and functional data is consistent with down-regulation of ENaC in response to high salt diet. However, this normal physiological adaptation did not occur in GR^βgeo/+^ mice: Instead, neither *Scnn1a* mRNA abundance (**Figure 6C**) nor benzamil-sensitive sodium reabsorption significantly reduced with high salt (**Figure 6D**) and this differential response to dietary salt between genotypes was highly significant (Effect of genotype *p* < 0.001).

## Discussion

We used GR^βgeo/+^ mice to model glucocorticoid resistance, assessing BP and renal sodium handling under basal and high salt conditions. The modest elevation of basal BP in GR^βgeo/+^ mice was amplified by high salt intake. Salt-sensitivity was associated with increased levels of deoxycorticosterone and corticosterone, an abnormal renal hemodynamic response and a failure of the distal nephron sodium to adapt to high salt intake.

### Corticosteroids and Salt-Sensitive Hypertension

Hypertension is a common feature of glucocorticoid excess, often attributed to increased mineralocorticoid bioactivity, which may occur through three possible routes. First, ACTH induces morphological and transcriptional changes in the adrenal gland that induce steroidogenesis in the *zona glomerulosa* (Mazzocchi et al., 1986; Menzies et al., 2017), although the effect on aldosterone production may only be transient (Dunbar et al., 2010). GR^βgeo/+^ mice have hypertrophied adrenal glands and an expanded *zona glomerulosa* but the elevated circulating aldosterone concentration reflected increased plasma renin activity, rather than high levels of ACTH (Michailidou et al., 2008). We found enhanced basal urinary excretion of aldosterone in GR^βgeo/+^ mice but the renin-angiotensin system modulated appropriately with high salt diet and was not a cause of salt-sensitive BP. Second, transcriptional responses resulting in corticosterone excess in mice increase production of the biosynthetic intermediate deoxycorticosterone (Dunbar et al., 2010). In our study, a high salt diet increased deoxycorticosterone excretion, to a greater extent in GR^βgeo/+^ mice. Deoxycorticosterone is a weak mineralocorticoid but in mice becomes biologically significant when the HPA axis is chronically activated (Mullins et al., 2009). Indeed, deoxycorticosterone acetate, in combination with high salt intake, is widely used to induce experimental hypertension in rodents (Basting and Lazartigues, 2017). Deoxycorticosterone may therefore contribute to salt-sensitive hypertension in the current study. Third, glucocorticoids can directly activate MR if levels exceed the inactivating capacity of 11βHSD2. We found no evidence for altered 11βHSD2 at the transcript level but expression of MR was significantly increased by high salt diet to a similar extent in both genotypes. This was an unexpected finding. One study has also found a positive relationship between dietary salt and MR expression in rat colon and, to a lesser extent, kidney (Norregaard et al., 2003), but another reports down-regulation of MR by high salt (Lienhard et al., 2012). We do not know why high salt induces MR expression but importantly in GR^βgeo/+^ mice the increased ratio of MR:11βHSD2 expression was also accompanied by a 4-fold increase in urinary corticosterone excretion, suggesting that glucocorticoids have the potential to act as mineralocorticoids in this genetic setting. Indeed, this concept is supported by the analysis of urinary steroid metabolites in patients with glucocorticoid resistance, which indicates that the 11βHSD2 barrier is breeched, allowing MR to be activated by cortisol (Bouligand et al., 2010). The expression of serum- and glucocorticoid-induced kinase-1 (sgk1) has been used to report MR activation in mice (e.g., Shibata et al., 2008). This was not measured in the current study since sgk1 is also stimulated by GR agonists (Alvarez de la Rosa et al., 2003) and would not discriminate unequivocally between MR and GR activation in this mouse model. Indeed, the hypertension of glucocorticoid excess can also involve GR pathways (Nieman et al., 1985; Bailey et al., 2009).

Our study also adds to a growing literature in rodents and humans showing that high salt intake enhances glucocorticoid bioactivity, either by increasing circulating steroid or by stimulating enzymatic intracellular amplification (Lewicka et al., 1998; Rakova et al., 2017). We do not know why high salt diet increases corticosterone. This was not a focus of our study and a systematic analysis of the relationship between dietary salt and HPA activity is currently lacking. Nevertheless, this positive relationship is exaggerated in both clinical and experimental models of salt-sensitive hypertension (Liu et al., 2008; Usukura et al., 2009; Bailey et al., 2011; Craigie et al., 2012; Huesler et al., 2013).

### Vascular Dysfunction and Salt-Sensitive Hypertension

Increased salt intake triggers a decrease in systemic vascular resistance in salt-resistant but not salt-sensitive humans and vascular dysfunction is proposed as a key mechanism of salt-sensitivity (Morris et al., 2016). Certainly, glucocorticoids influence vascular tone by potentiating the contractile response of resistance arteries to catecholamines and reducing vasodilatory responses to acetylcholine (Yang and Zhang, 2004). In our experiment, high-salt feeding to wild-type mice increased renal blood flow and reduced renal vascular resistance; this response was attenuated in GR^βgeo/+^ mice. A limitation of this study is that our measurements of renal blood flow were indirect, based on the clearance of *p*-aminohippurate rather than direct measurement by Doppler ultrasound. Nevertheless, vascular dysfunction may be an important, causative feature of salt-sensitivity in GR^βgeo/+^ mice. Certainly other studies suggest this as genetic deletion of the receptor in the endothelium increases basal blood pressure (Goodwin et al., 2011). Impaired vascular adaptation to high salt diet may also reflect MR activation, which is detrimental to vascular function in this setting (Ferrario and Schiffrin, 2015).

### Sodium Retention, Tubular Sodium Reabsorption and Salt-Sensitive Hypertension

The switch to high salt diet cause a transient sodium retention in GR^βgeo/+^ mice yet beyond this point, when neutral sodium balance was regained, we found a persistent enhancement of tubular sodium reabsorption. This may seem incompatible but restoration of sodium balance is of major homeostatic importance and will occur despite underlying tubular abnormalities that impair the acute pressure natriuresis response (Ivy and Bailey, 2014; Hall, 2016). Thus, sodium excretion can match intake over period of 24 h even if there is a reduced ability to excrete sodium rapidly. Such delayed attainment of balance impacts BP, disturbing normal circadian rhythm (Bankir et al., 2008) and causing salt-sensitivity (Staessen et al., 1993), as seen in GR^βgeo/+^ mice. To identify the molecular mechanism of altered tubular sodium transport, we focused on the major apical sodium transporters in the aldosterone-sensitive distal nephron, NCC and ENaC (Bailey, 2016), comparing measurements of mRNA with an *in vivo* assessment of transporter function. We did not measure protein abundance or distribution, a limitation to our study, but for ENaC, the mRNA and pharmacological data gave a consistent message: ENaC, which normally downregulates at both mRNA and protein levels with high salt intake (Udwan et al., 2017), did not downregulate in GR^βgeo/+^ mice despite reduced aldosterone. Sustained ENaC-mediated sodium reabsorption could be attributed to a salt-induced increase in deoxycorticosterone and/or corticosterone. Glucocorticoids can activate ENaC via GR (Schulz-Baldes et al., 2001; Bailey et al., 2009; Nguyen Dinh Cat et al., 2009; Craigie et al., 2012), but here it is more likely that the 11βHSD2 barrier is exceeded (Bouligand et al., 2010) and ENaC is activated via MR, as discussed above.

For NCC, mRNA and thiazide-sensitivity did not directly correlate but were directionally coherent. Thus, in GR^βgeo/+^ mice fed a high-salt diet, mRNA and thiazide-sensitive sodium transport were both higher than in GR^+/+^ mice. Why this occurs is not certain. High salt diet normally reduces total NCC protein and phosphorylation at site important for trafficking to the apical membrane of the distal convoluted tubule cell (Udwan et al., 2017). In GR^βgeo/+^ mice, thiazide-sensitive sodium reabsorption was not reduced by high salt diet but we do not know if this reflects an increase in the total NCC protein pool, as suggested by mRNA, and/or a change in the proportion of NCC that is phosphorylated. The distal convoluted tubule expresses MR and GR (Ackermann et al., 2010) but there is no expression of 11βHSD2 in the early part and only low levels toward the end of this segment (Hunter et al., 2015; Ivy et al., 2016). Chronic aldosterone excess can increase NCC activity (Ashek et al., 2012; Poulsen and Christensen, 2017) but this is secondary to mineralocorticoid-induced hypokalemia (Czogalla et al., 2016) and high-salt diet did not induce hypokalemia in GR^βgeo/+^ mice. A GR-mediated response is also possible since dexamethasone increases expression (Chen et al., 1994) and *in vivo* activity of NCC (Velazquez et al., 1996). However, given the reduced receptor expression in GR^βgeo/+^ mice, the importance of this pathway is uncertain.

In summary, this study shows that a global reduction in GR expression induces a pro-hypertensive relationship between blood pressure and dietary salt intake in mice, mostly likely reflecting MR activation by glucocorticoids. This provides mechanistic insight into the importance of genetic and environmental interactions in determining cardiovascular risk.

## Author Contributions

LE conducted some renal clearance experiments and the metabolic cage experiments, analyzed these experimental data and helped to revise the manuscript. JI conducted some renal clearance experiments, the radiotelemetry experiments, analyzed these experimental data and helped to draft and revise the manuscript. RM and RR performed qPCR experiments, analyzed these experimental data and commented on the manuscript during development. EAl-D performed urinary steroid analysis. PF, CK, and KC contributed to experimental design, discussion of data sets and development of the project concept and helped to draft and revise the manuscript. MB conducted some renal clearance experiments, performed statistical analysis, contributed to development of the project concept and experimental design, obtained research funding, drafted and revised the manuscript.

## Conflict of Interest Statement

The authors declare that the research was conducted in the absence of any commercial or financial relationships that could be construed as a potential conflict of interest.
